# Preoperative cardiac troponin I as a predictor of postoperative cardiac events in patients with end stage renal disease undergoing non-cardiac surgery

**DOI:** 10.1007/s00380-022-02159-z

**Published:** 2022-09-16

**Authors:** Bo Eun Park, Myung Hwan Bae, Yoon Jung Park, Hong Nyun Kim, Namkyun Kim, Se Yong Jang, Jang Hoon Lee, Dong Heon Yang, Hun Sik Park, Yongkeun Cho, Shung Chull Chae

**Affiliations:** Division of Cardiology, Department of Internal Medicine, Kyungpook National University Hospital, School of Medicine, 130, Dongdeok-ro, Jung-gu, Daegu, 41944 South Korea

**Keywords:** Cardiac troponin I, End stage renal disease, Surgery, Postoperative complications, Myocardial infarction, Risk assessment

## Abstract

We investigated if elevated cardiac troponin I (cTnI) serum levels before non-cardiac surgery were predictors of postoperative cardiac events in patients with end stage renal disease (ESRD) undergoing dialysis. In total, 703 consecutive patients with ESRD undergoing dialysis who underwent non-cardiac surgery were enrolled. Preoperative cTnI serum levels were measured at least once in all patients. The primary endpoint was defined as a composite of cardiac death, myocardial infarction (MI), and pulmonary edema during hospitalization or within 30 days after surgery in patients with a hospitalization longer than 30 days after surgery. Postoperative cardiac events occurred in 48 (6.8%) out of 703 patients (cardiac death 1, MI 18, and pulmonary edema 33). Diabetes mellitus (DM), previous ischemic heart disease, and congestive heart failure were more common in patients with postoperative cardiac events. Peak cTnI serum levels were higher in patients with postoperative cardiac event (180 ± 420 ng/L vs. 80 ± 190 ng/L, *p* = 0.008), and also elevated peak cTnI levels > 45 ng/L were more common in patients with postoperative cardiac events (66.8% vs. 30.5%, *p* < 0.001). Multivariate logistic regression analysis showed that DM (odds ratio [OR]  2.509, 95% confidence interval [CI] 1.178–5.345, *p* = 0.017) and serum peak cTnI levels ≥ 45 ng/L (OR 3.167, 95% CI 1.557–6.444, *p* = 0.001) were independent predictors for the primary outcome of cardiac death/MI/pulmonary edema. Moreover, cTnI levels ≥ 45 ng/L had an incremental prognostic value to the revised cardiac risk index (RCRI) (Chi-square = 23, *p* < 0.001), and to the combined RCRI and left ventricular ejection fraction (Chi-square = 12, *p* = 0.001). Elevated preoperative cTnI levels are predictors of postoperative cardiac events including cardiac death, MI, and pulmonary edema in patients with ESRD undergoing non-cardiac surgery.

## Introduction

Patients with chronic kidney disease (CKD) have a higher risk of cardiovascular (CV) disease than non-CKD patients, as CKD has been shown to have a high risk of arteriosclerosis progression [1]. Most patients also have other comorbidities such as hypertension and diabetes mellitus (DM) which are also risk factors for CV disease [1–3]. Elevated serum cardiac troponin I (cTnI) levels have been previously used to diagnose myocardial infarction (MI) and predict the risk of cardiac events [1–4]. However, serum cTnI levels are also increased during myocardial wall distension due to volume and pressure overloading from for example, decompensated heart failure, pulmonary thromboembolism, and extremely high blood pressure, symptoms which also occur in patients with CKD [4–6]. Therefore, when cTnI levels are increased during CKD, especially in dialysis patients, it is often difficult to interpret whether this is due to MI or CKD [7, 8].

CV complications after surgery have a significant impact on patient mortality and morbidity, therefore, the evaluation of postoperative CV events before surgery is vital [9]. Several methods have been proposed to predict postoperative CV events in preoperative patients; the revised cardiac risk index (RCRI) score is one such method [9]. Additionally, monitoring cTnI serum levels is sometimes used for high-risk patients, with some studies reporting elevated preoperative cTnI levels are related to postoperative CV events. However, few studies have reported if elevated preoperative cTnI levels can predict postoperative CV events in dialysis patients [10–13].

Therefore, we investigated if elevated cTnI serum levels before non-cardiac surgery could predict postoperative cardiac events in patients with end stage renal disease (ESRD) undergoing dialysis.

## Methods

### Study population

From all patients with end stage renal disease (ESRD) undergoing dialysis at the Kyungpook National University Hospital between January 2015 and December 2019, we included 703 consecutive patients who underwent non-cardiac surgery. The following patients were excluded; those diagnosed with myocardial infarction (MI) within 30 days before surgery, and those with sepsis. The study was approved by the Institutional Review Board of Kyungpook National University Hospital (No: 2021-06-004). Informed consent was waived by the board.

### Data collection

Patient information was obtained from medical chart. Patient demographics included age, sex, and body mass index (BMI). Blood samples were taken at admission. Cardiovascular (CV) risk factors included hypertension, diabetes mellitus (DM), a previous history of ischemic heart disease (IHD)/congestive haert failure (CHF)/cerebrovascular disease (CVD), and current smoking status. Echocardiographic results were based on examinations performed on the closest date prior to surgery. During echocardiography, the left ventricular ejection fraction (LVEF, %), left ventricular end diastolic dimension (LVEDD, mm), left atrium anteroposterior diameter (mm), and early diastolic mitral inflow velocity/early diastolic mitral annular tissue velocity (E/E') were measured. Electrocardiography (ECG) results were based on examinations at admission. ECG findings included the presence of atrial fibrillation and ST-T changes, and QRS duration (ms). In addition, patient anesthesia type, the risk of surgery, and the revised cardiac risk index (RCRI) scores were included, and the risk of major cardiac adverse events according to the type of surgery was classified into high (> 5%), intermediate (1 ~ 5%), and low risk (< 1%) [14, 15].

### Measurement of cardiac troponin I

For all patients, serum cardiac troponin I (cTnI) (ng/L) levels were evaluated at admission or within at least 2 weeks prior to surgery. For patients with abnormal cTnI values, assays were performed repeatedly. Serum cTnI levels were measured using the Dimension Vista system (DIMENSION VISTA^®^ CTNI CARDIAC TROPONIN I FLEX^®^: Siemens Healthcare Diagnostics Inc., NY, USA). The limit of quantitation was determined at 40 ng/L and corresponded to a coefficient of variation (CV) of 10%. In a study of 199 serum samples from apparently healthy individuals, the upper limit of the 99th percentile for the method was 45 ng/L. The reportable range of the assay was 15–40,000 ng/L.

### Clinical outcomes

The primary outcome was defined as cardiac events during hospitalization or within 30 days after surgery in patients with a hospitalization longer than 30 days after surgery. Cardiac events consisted of cardiac death, MI, and pulmonary edema. Secondary outcomes consisted of cardiac death and MI. Acute MI was defined when there is acute myocardial injury with clinical evidence of acute myocardial ischemia and with detection of a rise and/or fall of cTnI values with at least one value above the 99th percentile upper reference limit and at least one of the following: symptoms of myocardial ischemia; new ischemic electrocardiogram changes; development of pathological Q waves; imaging evidence of new loss of viable myocardium or new regional wall motion abnormality in a pattern consistent with an ischemic etiology; identification of a coronary thrombus by angiography or autopsy [11]. A pulmonary edema diagnosis required one or more of the following conditions: development of symptoms or signs of pulmonary edema and evidence of abnormal findings from chest radiography [12].

### Statistical analysis

Data were expressed as the mean ± standard deviation, and percentages for continuous and categorical variables. Comparisons between baseline variables were performed using student *t* tests and Chi-square tests for continuous and categorical variables, respectively. All *p*-values were two-sided, and a *p* < 0.05 value was considered statistically significant. Multivariate logistic regression analysis was used to identify independent predictors of cardiac events. Incremental factors added to the model at each step were considered significant when differences in log-likelihoods associated with models corresponded to *p* < 0.05. Receiver operating characteristic (ROC) curve analyses were performed to determine cut-off values for predicting cardiac events and estimated ROC curves and compared areas under curves (with 95% confidence interval [CI]) in corresponding logistic models. All statistical analyses were performed using IBM SPSS version 20.0 (IBM Corp., Armonk, NY, USA) and MedCalc version 19.8 (MedCalc Software, Ostend, Belgium).

## Results

Baseline characteristics of the 703 patients (mean age; 61.4 ± 14.7 years; 425 males [60.5%]) are shown (Table 1). Hypertension (80.9%) and diabetes mellitus (DM) (59%) were common comorbidities. We observed that 115 patients (16.4%) had experienced previous ischemic heart disease (IHD) and 93 (13.2%) and 83 patients (11.8%) had a previous history of congestive heart failure (CHF) and cerebrovascular disease (CVD), respectively. The mean left ventricular ejection fraction (LVEF) was 54.1% ± 10.4%, mean left ventricular end diastolic dimension (LVEDD) was 48.7 ± 7.2 mm, and mean early diastolic mitral inflow velocity/early diastolic mitral annular tissue velocity (E/E') was 15.0 ± 6.7. In terms of laboratory findings, patients with peak cardiac troponin I (cTnI) serum levels ≥ 45 ng/L totaled 233 (33.1%): the mean peak cTnI serum level was 90 ± 200 ng/L. Peak cTnI level positively correlated with the LVEDD (*r* = 0.079, *p* = 0.037) and E/E' (*r* = 0.095, *p* = 0.011), but there was no correlation between peak cTnI and LVEF (*r* = −0.054, *p* = 0.154).

**Table 1:** Baseline characteristics according to the presence or absence of cardiac events

	All patients (*n* = 703)	Cardiac events			*P* value
		Yes (*n*= 48)		No (*n* = 655)	
Demographics					
Age (year)	61.4 ± 14.7	64.5 ± 14.2		61.2 ± 14.7	0.132
Male (%)	425 (60.5)	31 (64.6)		394 (60.2)	0.545
Body mass index (kg/m^2^)	22.6 ± 3.6	22.8 ± 3.9		22.5 ± 3.6	0.626
Risk factors and comorbidities					
Hypertension (%)	569 (80.9)	39 (81.2)		530 (80.9)	0.955
Diabetes, all (%)	415 (59.0)	37 (77.1)		378 (57.7)	0.008
Diabetes, insulin (%)	155 (22.0)	13 (27.1)		142 (21.7)	0.383
Previous IHD (%)	115 (16.4)	19 (39.6)		96 (14.7)	< 0.001
Previous CHF (%)	93 (13.2)	11 (22.9)		82 (12.5)	0.040
Previous CVD (%)	83 (11.8)	8 (16.7)		75 (11.5)	0.280
Current smoking (%)	102 (14.5)	7 (14.6)		95 (14.5)	0.998
Peak cardiac troponin I (ng/L)	90 ± 200	180 ± 240		80 ± 190	0.008
Peak cardiac troponin I ≥ 45 ng/L (%)	233 (33.1)	33 (68.8)		200 (30.5)	< 0.001
Echocardiography					
LVEF (%)	54.1 ± 10.4	47.0 ± 15.1		54.7 ± 9.8	0.001
LVEDD (mm)	48.7 ± 7.2	51.5 ± 6.7		48.4 ± 7.2	0.006
E/E'	15.0 ± 6.7	19.0 ± 9.5		14.7 ± 6.4	0.007
Electrocardiography					
Atrial fibrillation (%)	33 (4.8)	3 (6.2)		30 (4.7)	0.493
QRS duration (ms)	96.3 ± 20.6	98.7 ± 23.4		96.6 ± 23.8	0.551
ST-T changes (%)	221 (31.9)	25 (52.1)		196 (30.4)	0.002
General anesthesia (%)	255 (37.7)	11 (61.1)		254 (37.1)	0.038
Surgical risk according to type of surgery					
High (%)	71 (10.1)	4 (8.3)		67 (10.2)	0.152
Intermediate (%)	302 (43.0)	16 (33.3)		286 (43.7)	
Low (%)	330 (46.9)	28 (58.3)		302 (46.1)	
RCRI score	1.7 ± 0.8	2.2 ± 0.9		1.7 ± 0.8	< 0.001

*IHD* ischemic heart disease, *CHF* congestive heart failure, *CVD* cerebrovascular disease, *LVEF* left ventricular ejection fraction, *LVEDD* left ventricular end diastolic dimension, *E/E*' early diastolic mitral inflow velocity/early diastolic mitral annular tissue velocity, *RCRI* revised cardiac risk index

Cardiac events occurred in 48 (6.8%) patients (cardiac death; 1 patient, myocardial infarction (MI); 18 patients, pulmonary edema; 33 patients). DM, previous IHD, and CHF were more common in patients with postoperative cardiac events. The mean peak cTnI serum level was 9 ± 200 ng/L, and peak cTnI levels were higher in patients with cardiac events (180 ± 240 vs. 80 ± 190, *p* = 0.008). Also, peak cTnI levels ≥ 45 ng/L were more common in patients with cardiac events (66.8% vs. 30.5%, *p* < 0.001). Echocardiographic results showed lower LVEF (47.0% ± 15.1% vs. 54.7% ± 9.8%, *p* = 0.001), higher LVEDD (51.5 ± 6.7 mm vs. 48.4 ± 7.2 mm, *p* = 0.006), and higher E/E' (19.0 ± 9.5 vs. 14.7 ± 6.4, *p* = 0.007) in patients with cardiac events. Also, patients with cardiac events showed a greater proportion of ST-T changes during 12-lead electrocardiography (ECG) than those without cardiac events (52.1% vs. 30.4%, *p* = 0.002). In addition, the general anesthesia rate was higher in patients with cardiac events (61.1% vs. 37.1%, *p* = 0.038) and the revised cardiac risk index (RCRI) scores were also higher in patients with cardiac events (2.2 ± 0.9 vs. 1.7 ± 0.8, *p* < 0.001). The groups with high, intermediate, and low risk of cardiac major adverse cardiac event according to the type of surgery were 10.1% (*n* = 71), 43.0% (*n* = 302), and 46.9% (*n* = 330), respectively. However, there was no statistical difference in the incidence of postoperative cardiac events between the three groups. Also we observed no between-group differences regarding age, sex, body mass index (BMI), and comorbidities such as hypertension, previous CVD, and smoking status.

**Fig. 1:**
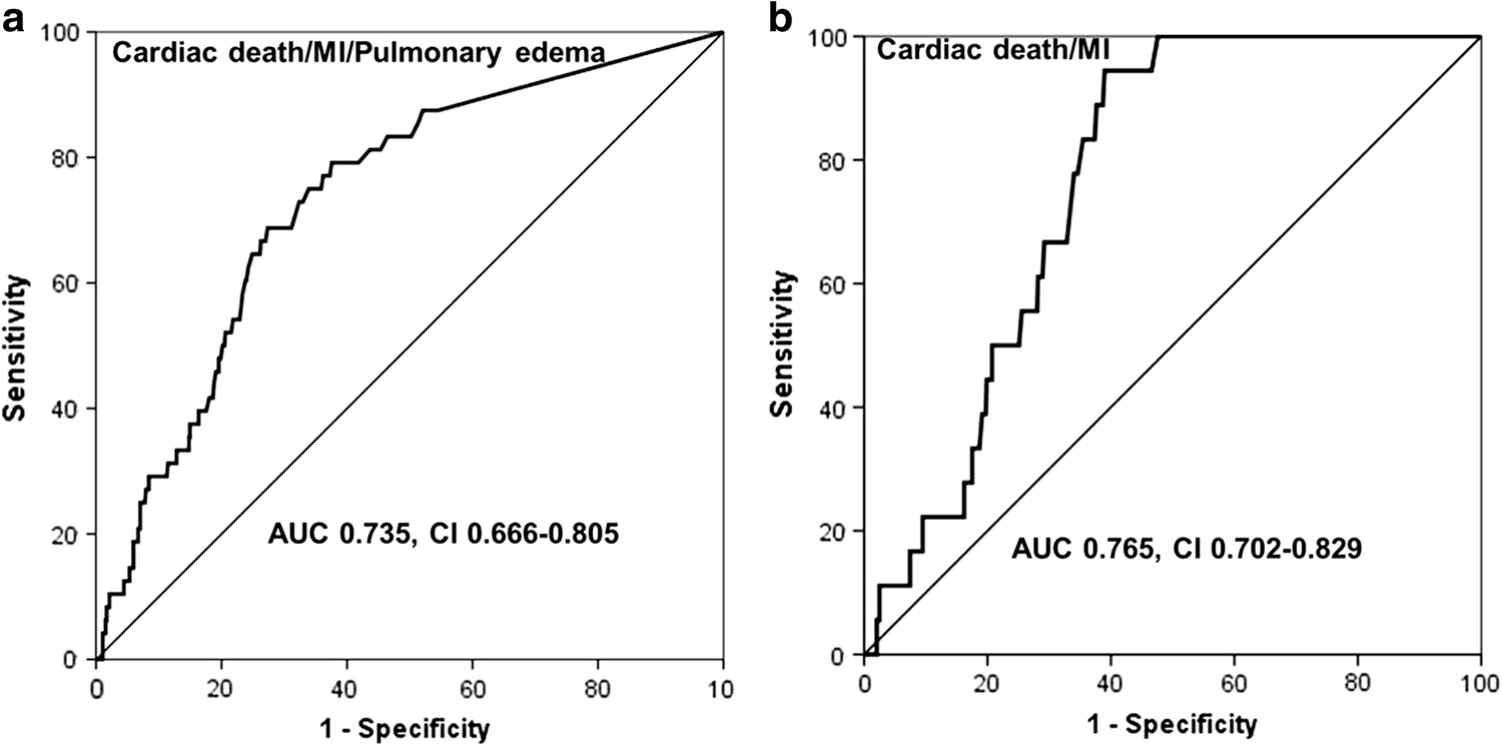
**a** The area under the ROC curve for predicting postoperative cardiac events (cardiac death/ myocardial infarction MI/pulmonary edema) for preoperative cTnI. **b** The area under the ROC curve for predicting postoperative cardiac events (cardiac death/MI) for preoperative cTnI. *ROC* receiver operating characteristics; *MI* myocardial infarction; *cTnI* cardiac troponin I

The area under the receiver operating characteristic (ROC) curves for prediction of postoperative cardiac events (cardiac death/MI/pulmonary edema) and cardiac death/MI were 0.735 (95% confidence interval [CI] 0.666–0.805) and 0.765 (95% CI 0.702–0.829) for preoperative cTnI, respectively (Fig. 1). When groups were classified based on cTnI ≥ 45 ng/L levels, the frequency of postoperative MI (*p* = 0.009) and pulmonary edema (*p* < 0.001) was higher in patients with peak cTnI ≥ 45 ng/L levels. Also, the composite of cardiac death, MI, and pulmonary edema (*p* < 0.001) and the composite of cardiac death and MI (*p* = 0.009) were also high. However, no statistical differences in cardiac death were observed according to the presence or absence of elevated peak cTnI levels (*p* = 0.543) (Table 2).

**Table 2:** Cardiac events according to the levels of preoperative peak cardiac troponin I

	cTnI (1–44 ng/L) (*n* = 470)	cTnI (≥ 45 ng/L) (*n* = 233)	*p* value
Cardiac death/MI (%)	6 (1.3)	12 (5.2)	0.004
Cardiac death (%)	0 (0)	1 (0.4)	0.331
Myocardial infarction (%)	6 (1.3)	12 (5.2)	0.004
Cardiac death/MI/pulmonary edema (%)	15 (3.2)	33 (14.2)	< 0.001
Pulmonary edema (%)	11 (2.3)	22 (9.4)	< 0.001

*cTnI* cardiac troponin I, *MI* myocardial infarction

**Fig. 2:**
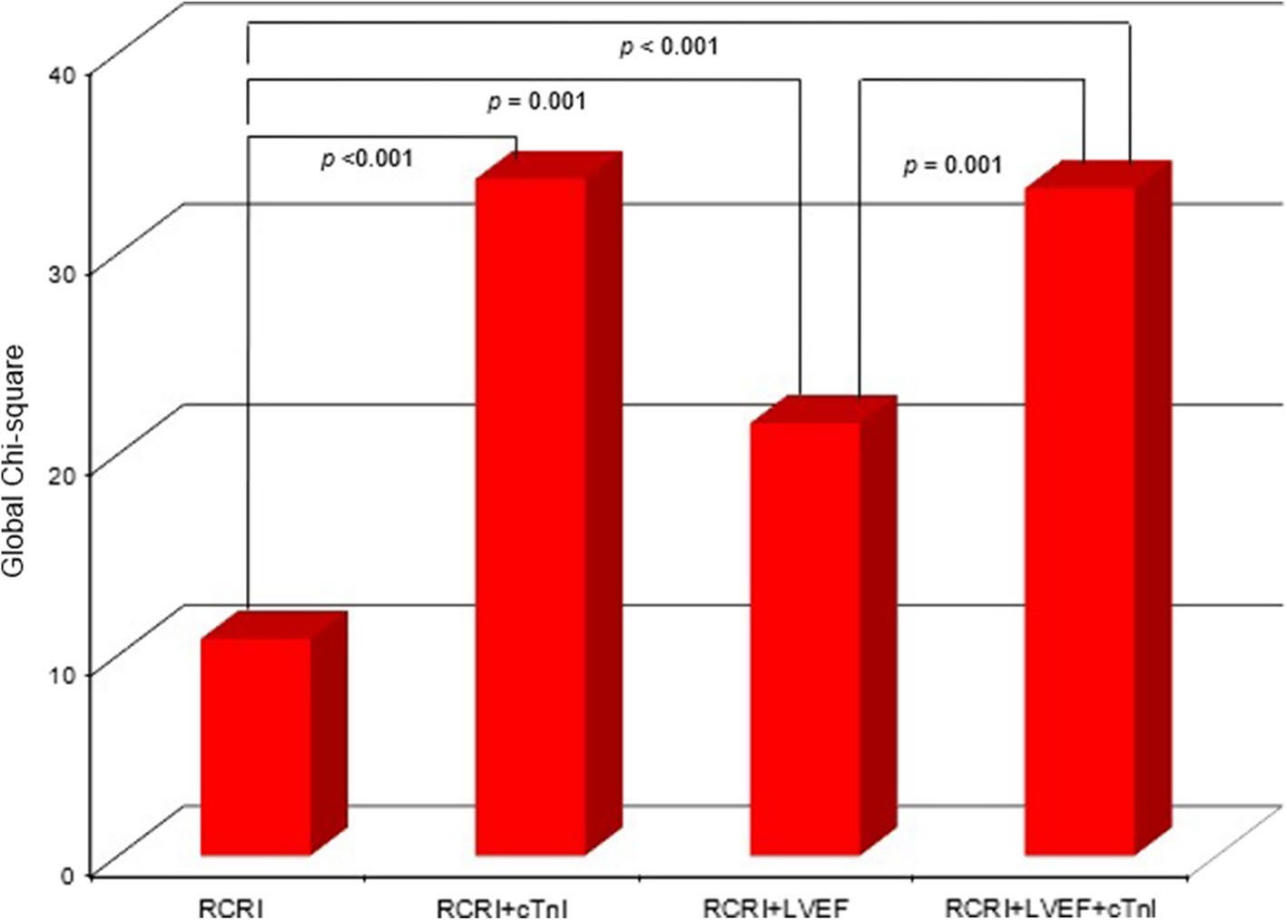
Incremental prognostic value of sequential RCRI, cTnI, and LVEF. Global Chi-square on the *Y* axis indicates the incremental prognostic value of sequential RCRI, cTnI, and LVEF. *RCRI* revised cardiac risk index; *cTnI* cardiac troponin I; *LVEF* left ventricular ejection fraction

Multivariate logistic regression analyses showed that DM (OR 2.509, 95% CI 1.178–5.345, *p* = 0.017) and peak cTnI ≥ 45 ng/L levels (OR 3.167, 95% CI 1.557–6.444, *p* = 0.001) were independent predictors for the primary outcome; cardiac death/MI/pulmonary edema (Table 3). Moreover, cTnI ≥ 45 ng/L levels had an incremental prognostic value for RCRI scores (Chi-square = 23, *p* < 0.001) and for combined RCRI and LVEF data (Chi-square = 12, *p* = 0.001) (Fig. 2).

**Table 3:** Multivariate logistic regression analysis for predicting cardiac events

	Cardiac events (death/MI/pulmonary edema)			Cardiac events (death/MI)		
	Odds ratio	95% CI	*p* value	Odds ratio	95% CI	*p* value
Diabetes	2.509	1.178–5.345	0.017	5.438	1.147–25.767	0.033
Previous ischemic heart disease	1.906	0.941–3.861	0.073	1.374	0.423–4.458	0.597
Previous congestive heart failure	0.656	0.259–1.660	0.373	1.180	0.305–4.570	0.810
Left ventricular ejection fraction	0.968	0.935–1.002	0.068	0.937	0.888–0.990	0.020
Left ventricular end diastolic dimension	1.149	0.683–1.934	0.601	0.882	0.368–2.113	0.778
ST-T changes on the ECG	1.391	0.710–2.726	0.336	2.019	0.645–6.326	0.228
General anesthesia	1.312	0.655–2.627	0.443	5.575	1.822–17.060	0.003
Peak cardiac troponin I ≥ 45 ng/L (%)	3.167	1.557–6.444	0.001	2.175	0.651–7.274	0.207

*MI* myocardial infarction, *CI* confidence interval, *ECG* electrocardiography, *cTnI* cardiac troponin I

**Fig. 3:**
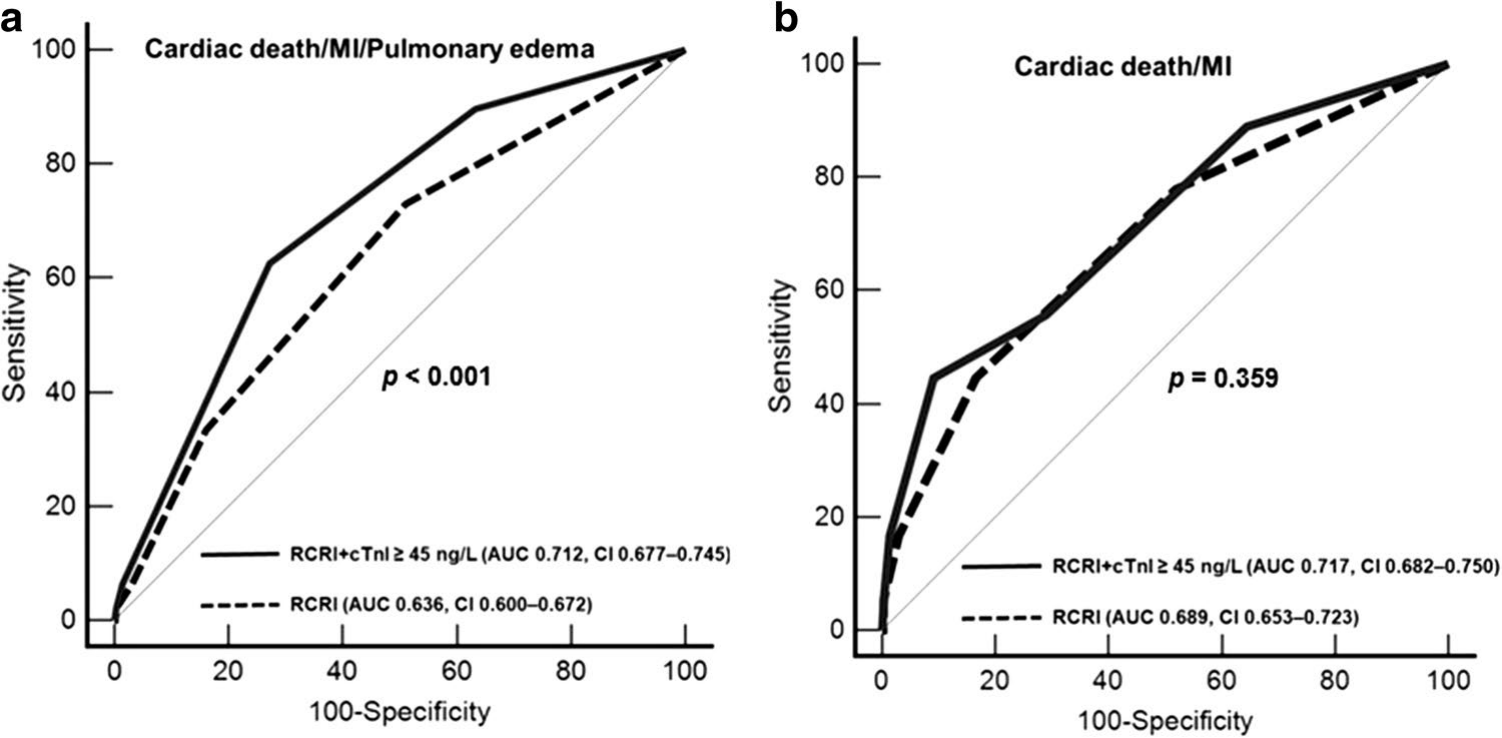
**a** The area under the ROC curve for predicting cardiac death, MI, and pulmonary edema for the RCRI and reclassifying the RCRI with the sum of the RCRI score and cTnI ≥ 45 ng/L as 1 point. **b** The area under the ROC curve for predicting cardiac death and MI for the RCRI, and the RCRI with the sum of the RCRI score and cTnI ≥ 45 ng/L as 1 point. *ROC* receiver operating characteristics; *MI* myocardial infarction; *RCRI* revised cardiac risk index; *cTnI* cardiac troponin I

When the RCRI score was reclassified by summing the RCRI and peak cTnI ≥ 45 ng/L, The area under the ROC curve for predicting postoperative cardiac events (cardiac death/MI/pulmonary edema) was 0.636 (95% CI 0.600–0.672) for RCRI. Adding peak cTnI ≥ 45 ng/L to RCRI significantly increased the area under the curve to 0.712 (95% CI 0.677–0.745, *p* < 0.001) (Fig. 3a). However, the area under the ROC curve for predicting the cardiac death and MI was not significant differences between the RCRI and the combination of RCRI and peak cTnI ≥ 45 ng/L (*p* = 0.359) (Fig. 3b).

**Fig. 4:**
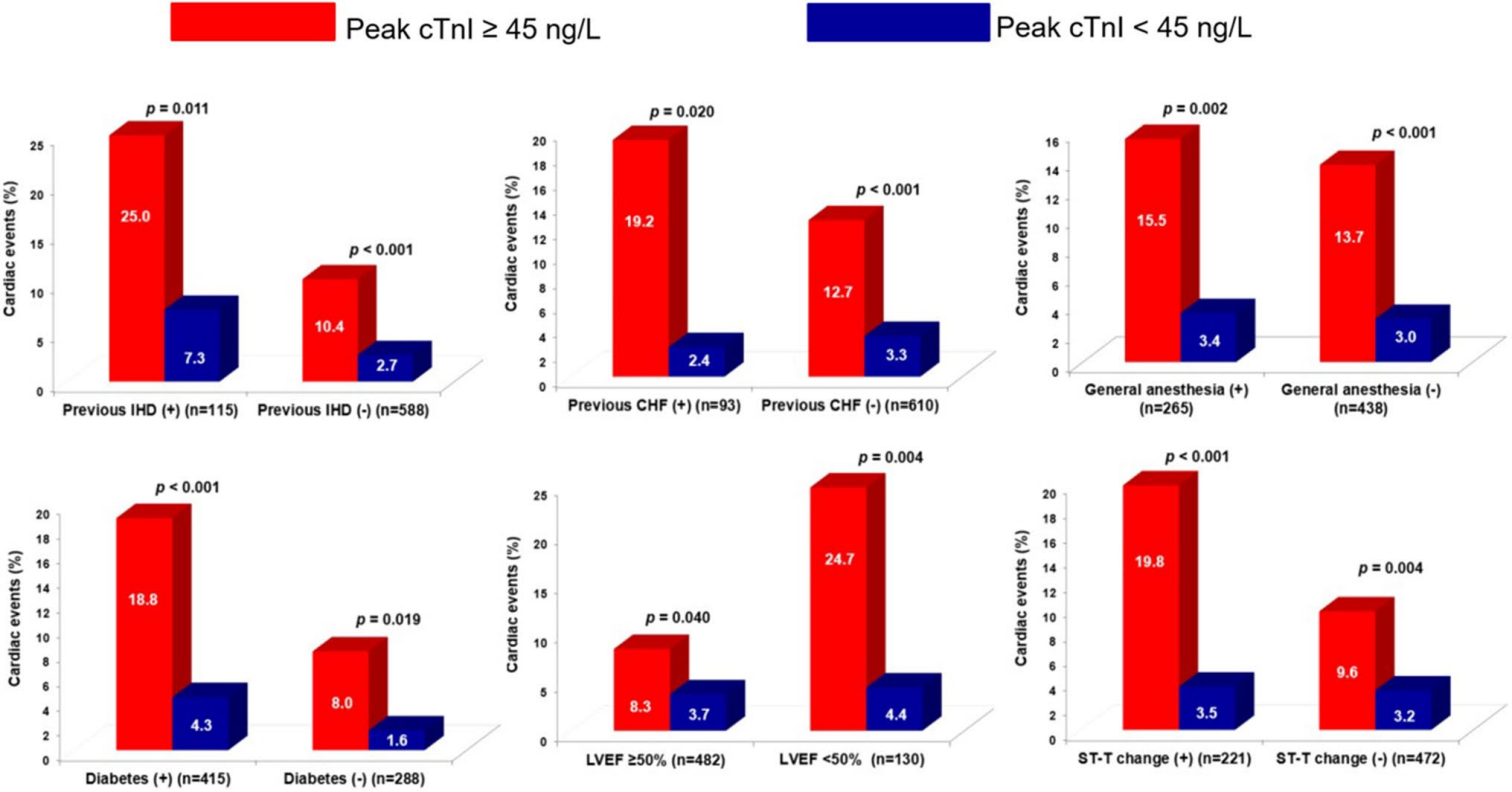
Cardiac events in patients with elevated preoperative cardiac troponin I levels compared to those without, regardless of diabetes mellitus, left ventricular ejection fraction, ST-T changes on electrocardiography, general anesthesia, previous ischemic heart disease and congestive heart failure histories

In subgroup analysis, cardiac events were significantly higher in patients with elevated preoperative cTnI levels when compared to those without, regardless of DM, LVEF, ST-T changes by ECG, general anesthesia, and previous IHD and CHF histories (all *p* for interaction > 0.05) (Fig. 4).

## Discussion

Our main findings indicated that elevated preoperative cardiac troponin I (cTnI) levels predicted postoperative cardiac events, including cardiac death, myocardial infarction (MI), and pulmonary edema in patients with end stage renal disease (ESRD) undergoing non-cardiac surgery. In addition, combined the revised cardiac risk index (RCRI) and cTnI scores were more powerful than RCRI scores alone for postoperative cardiac risk stratification.

Methods predicting postoperative cardiac events using appropriate preoperative evaluations, e.g., risk stratification, have been comprehensively studied [9, 14–16]. The RCRI was designed to predict postoperative MI, pulmonary edema, ventricular fibrillation or cardiac arrest, and complete heart block. The RCRI model for cardiac perioperative risk stratification has been recommended by several guidelines, which also suggested preoperative cardiac troponin measurements may be helpful in high-risk patients, such as those with chronic kidney disease (CKD).

Several studies have investigated if preoperative cardiac troponin levels could predict postoperative cardiac complications; Weber et al*.* observed that high sensitivity cardiac troponin T levels helped predict prognosis after non-cardiac surgery [17–19]. In addition, elevated preoperative cTnI levels were associated with mortality and major adverse cardiac events (MACEs) after non-cardiac surgery [18, 20–24]. Similarly, a meta-analysis also reported preoperative elevated levels of cardiac troponin for the risk assessment of postoperative MACEs and mortality [25]. It was also shown that preoperatively increased cardiac troponin could be used to indicate postoperative MACEs and mortality. However, cTnI may be elevated in patients with several diseases or conditions, without ischemic heart disease (IHD). Most of the studies so far have focused on the general population. The general population and patients with ESRD may have different causes of elevated cTnI. Therefore, there is a limitation in applying the results of the general population study to these patients who are likely to have increased cTnI due to other reasons.

With our aging society, the number of patients with CKD is increasing year on year [26]. These patients experience more cardiovascular (CV) comorbidities than the general population and are at a higher risk of cardiac complication after non-cardiac surgery [27]. Moreover, patients with ESRD on dialysis frequently experience cTnI elevation without cardiac symptoms, eg. chest pain, dyspnea, etc. [28]. Therefore, the relationship between preoperative cTnI levels and postoperative cardiac events in previous general population studies has been proven to some extent, it is not reasonable to apply these results to CKD patients and questionable whether preoperative cTnI elevation is helpful in predicting cardiac event after non-cardiac surgery in patients with ESRD on dialysis. Several previous studies have focused on the relationship between cTnI levels and postoperative mortality in patients with ESRD, however, they were limited to relationships between elevated postoperative cTnI and patient prognoses [5–13]. The role of cTnI as a predictor of postoperative cardiac events in patients with ESRD remains unclear.

In this study, 33% of patients with ESRD had elevated cTnI levels before surgery; this potentially was a useful predictor of postoperative cardiac events. In addition, preoperative cTnI levels had an incremental value for the RCRI, an accepted clinical risk score [15]. Patients with ESRD on dialysis are a high-risk group for CV disease, with a very high risk of cardiac events after surgery. Therefore, preoperative risk assessments for this group are more critical than for the general population. However, sufficient preoperative risk assessments are often difficult to perform because the risk of the preoperative examination, eg. myocardial stress test is high. The cTnI assay is easily facilitated by a relatively simple blood test and is useful for evaluating a patient’s surgical risk without additional patient risk. Therefore, we propose cTnI is useful for predicting high-risk cardiac events after surgery in patients with ESRD.

The result that elevated cTnI did not predict postoperative fatal cardiac events, such as death/MI, except pulmonary edema, suggests that elevation of cTnI may have been caused by volume and/or pressure overload in addition to myocardial ischemia in dialysis patients. However, since cardiac events due to volume and/or pressure overload in dialysis patients can also lead to increased mortality and prolonged hospitalization, The results of our study demonstrating that elevation of preoperative cTnI can predict the occurrence of postoperative cardiac events, including pulmonary edema, are still meaningful.

Our study had limitations. Firstly, as it was retrospective in nature, there may be variables that can affect the results. Secondly, in most patients, cTnI testing was performed on the day of admission for surgery. Therefore, the relationship between the time of blood collection and the time of dialysis differed for each patient. Although cTnI may have increased due to volume overloading before dialysis, cTnI levels were normal in 67% of patients in our study. In addition, considering that most of the patients with elevated cTnI had repeated blood tests, we think that the timing of blood tests has insignificant effect on the results of our study. Thirdly, The cTnI used in our study was not the recently used high-sensitive cTnI. Therefore, there is a limit to applying the results of our study to high-sensitive cTnI. Finally, postoperative cTnI levels were not measured in all patients, so we may have missed a hidden cardiac event or a silent MI. However, postoperative cTnI measurements in all patients were not suitable in clinical practice, and most postoperative cTnI measurements were performed in patients with symptomatic or unstable vital signs. Therefore, it is thought that the effect on the study results was negligible.

In conclusion, elevated preoperative cTnI levels are predictors of postoperative cardiac events, including cardiac death, MI, and pulmonary edema in patients with ESRD undergoing non-cardiac surgery. Thus, the addition of cTnI measurements from relatively easy blood tests could help clinicians assess postoperative cardiac risks events in patients with ESRD.
